# Metformin and temozolomide act synergistically to inhibit growth of glioma cells and glioma stem cells *in vitro* and *in vivo*

**DOI:** 10.18632/oncotarget.5405

**Published:** 2015-09-26

**Authors:** Zhiyun Yu, Gang Zhao, Guifang Xie, Liyan Zhao, Yong Chen, Hongquan Yu, Zhonghua Zhang, Cai Li, Yunqian Li

**Affiliations:** ^1^ Department of Neurosurgery, First Hospital of Jilin University, Changchun, China; ^2^ Department of Obstetrics and Gynecology, First Hospital of Jilin University, Changchun, China; ^3^ Department of Clinical laboratory, Second Hospital of Jilin University, Changchun, China; ^4^ Department of Experimental Pharmacology and Toxicology, School of Pharmacy, Jilin University, Changchun, China

**Keywords:** AMPK, AKT-mTOR, glioma stem cell, metformin, temozolomide

## Abstract

Glioblastoma (GBM) is the most frequent and aggressive brain tumor in adults. In spite of advances in diagnosis and therapy, the prognosis of patients with GBM has remained dismal. The fast recurrence and multi-drug resistance are some of the key challenges in combating brain tumors. Glioma stem cells (GSCs) which are considered the source of relapse and chemoresistance, the need for more effective therapeutic options is overwhelming. In our present work, we found that combined treatment with temozolomide (TMZ) and metformin (MET) synergistically inhibited proliferation and induced apoptosis in both glioma cells and GSCs. Combination of TMZ and MET significantly reduced the secondary gliosphere formation and expansion of GSCs. We first demonstrated that MET effectively inhibited the AKT activation induced by TMZ, and a combination of both drugs led to enhanced reduction of mTOR, 4EBP1 and S6K phosphorylation. In addition, the combination of the two drugs was accompanied with a powerful AMP-activated protein kinase (AMPK) activation, while this pathway is not determinant. Xenografts performed in nude mice demonstrate *in vivo* demonstrated that combined treatment significantly reduced tumor growth rates and prolonged median survival of tumor-bearing mice. In conclusion, TMZ in combination with MET synergistically inhibits the GSCs proliferation through downregulation of AKT-mTOR signaling pathway. The combined treatment of two drugs inhibits GSCs self-renewal capability and partly eliminates GSCs *in vitro* and *in vivo*. This combined treatment could be a promising option for patients with advanced GBM.

## INTRODUCTION

Glioblastoma (GBM) is the most common and devastating primary malignant intracranial tumor in adults. The current standard of care for newly diagnosed GBM is surgical resection followed by radiotherapy plus concomitant and adjuvant temozolomide (TMZ) [1]. The prognosis is still relatively poor with a median overall survival is only 14.6 months, median progression free-survival is 6.9 months and 5 year survival rate only 9.8% after diagnosis [1, 2]. The fast recurrence and multi-drug resistance are some of the key challenges in combating brain tumors, control of the cancer stem cell population is considered key to realizing the long-term survival of GBM patients [3].

TMZ is the major chemotherapeutic drug used clinically in the treatment of GBM. The primary path leading to TMZ-induced cell death is formation of O-6-methylguanine and apoptotic signaling triggered by O-6-methyl G:T mispairs [4]. Recent studies suggested that one of the cytotoxic mechanisms of TMZ on GBM goes through an AMPK activation step [5]. Accumulating evidence shows that GBM frequently displays hyperactivation of the AKT pathway [6–8] and endogenous AKT kinase activity can be activated in response to clinically relevant concentrations of TMZ [9, 10]. Once activated, AKT phosphorylates several substrates involved in various cellular processes including cell proliferation, survival, growth and metabolism [7, 11]. AKT activation is also correlated with the increased tumorigenicity, invasiveness and stemness [7]. It has demonstrated that cancer stem cells are preferentially sensitive to an inhibitor of AKT [8, 12] and down-regulation of the AKT pathway can enhance the cytotoxicity of TMZ [9, 13, 14].

Epidemiologic data have revealed that MET, a first-line treatment for type-2 diabetes, can reduce cancer incidence and mortality in certain cancers [15, 16], and increase the number of breast carcinoma patients obtaining complete response to neo-adjuvant therapy [17]. Interestingly, studies have shown that metformin enhances antitumor efficiency of chemotherapeutic agents *in vitro* and *in vivo* [18–20]. Recently, MET has been shown to selectively kill cancer stem cells [21–24] with minor adverse effects [25]. Its mechanism of antiproliferative mainly by activating AMPK [22, 23] and inhibiting the activity of AKT [24, 26]. On these bases, several clinical trials are underway [27, 28]. Based on these preclinical studies it has been proposed that the combination of MET and TMZ may be an effective treatment for some cancer types. Recently, Sesen *et al* [29] have proved that MET treatment in combination with TMZ induces a synergistic anti-tumoral response in glioma cell lines, however, the mechanism has not been fully elucidated. Furthermore, whether MET can potentiate the cytotoxicity of TMZ for GSCs is still scantly explored.

In the present study, we first demonstrated that the combination treatment of TMZ and MET synergistically inhibited the growth and induced cell apoptosis in both U87 GSCs and U251GSCs with accompanying enhanced reduction of mTOR, S6K and 4EBP1 signaling. TMZ induced AKT activation also was effectively inhibited by MET. Although a combination of the two drugs was associated with a powerful AMPK activation, but it is not determinant. This synergy was confirmed *in vivo* by demonstrating significant reduction in tumor burden and significantly prolonged median survival of tumor-bearing mice after combined drug treatment as compared with treatment with either drug alone.

## RESULTS

### Isolation, characterization and differentiation of GSCs isolated from U87 and U251 glioma cell lines

**Figure 1:**
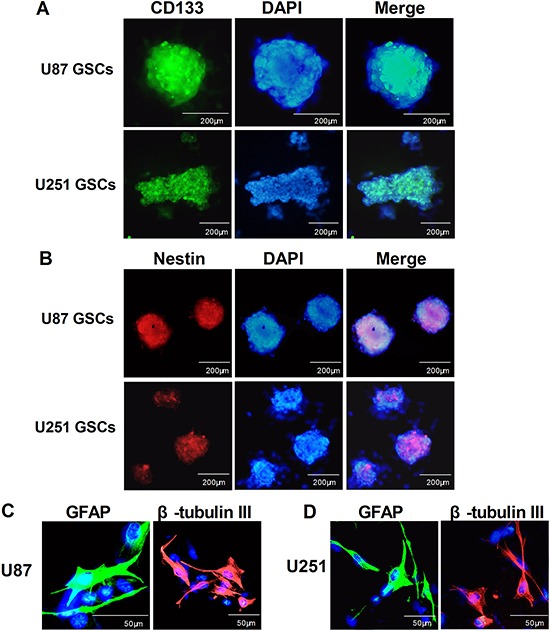
Identification, characterization and differentiation GSCs After primary spheres formation was noted, the primary gliospheres were dissociated and sub-cultured in NBM with B27, N2, glutaMAX,2 μg/ml heparin and 20 ng/ml EGF+bFGF for 7 days, immunocytochemical staining of the cancer stem cell marker CD133 **A.** and the neural progenitor markers nestin **B.** in U87 GSCs and U251 GSCs. For immunostaining of differentiated tumor cells, gliospheres were tanslated to DMEM with 10% FBS for another 7 days, then the U87 and U251 glioma cells immunostained with β-tubulin-III and GFAP **C.** The fluorescent signals were detected and photographed (× 200) with a fluorescence microscope (Olympus IX51,Japan).

**Figure 2:**
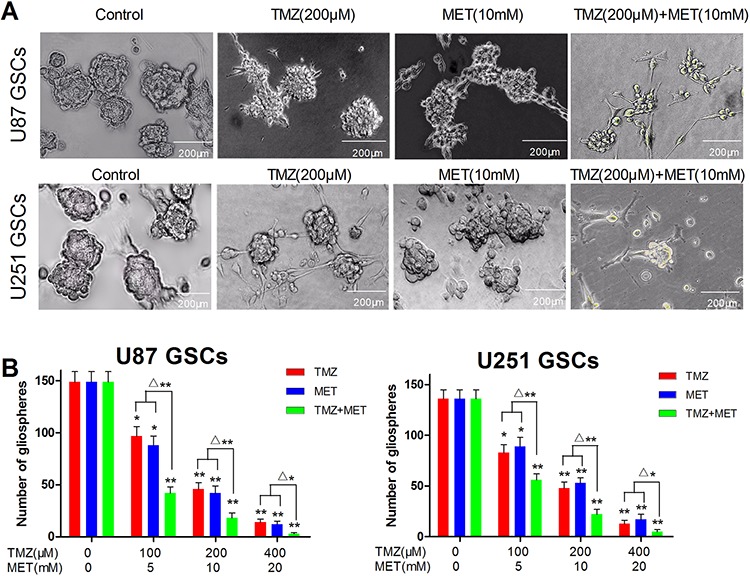
TMZ and MET combined to inhibit GSCs self-renewal and inhibit gliosphere formation and expansion U87 GSCs and U251 GSCs were cultured in NBM in the absence or presence of TMZ (100–400 μM), MET (5–20 mM) or TMZ+MET for 7 days and the number of gliospheres photographed (**A.** × 200) and counted **B.** under IX51 Olympus microscope. Results are representative of three independent experiments (*P < 0.05, **P < 0.01, compared with control group; ^Δ^*P < 0.05, ^Δ^**P < 0.01, TMZ+MET *vs* single drug).

U87 and U251 gliospheres cultures were characterized for recognized GSC signatures: self-renewal, neural stem cell marker expression and differentiation. The self-renewing capacity of the tumor spheres was assayed by dissociation of primary tumor spheres. When self-renewal capacity was compared among tumor subtypes at a plating density of 5 × 10^3^ cells/well, U87 were found to generate a greater mean number of secondary tumor spheres(151 ± 5) compared with U251(136 ± 9) (Figure 2). The cells within the sphere were positive to neural stem cell markers CD133 (Figure 1A) and nestin (Figure 1B), and lack of immunoreactivity for markers of differentiated neural cell types such as GFAP for astrocytes and β-tubulin III for neurons. The assay of multi-lineage differentiation capacity of cells within the sphere was demonstrated by culturing the cells in differentiation-inducing culture medium (DMEM+10% FBS) for 7 days. These cells lost expression of CD133 and nestin when subjected to differentiating conditions, showed typical morphological differentiation towards neuronal and astrocytic lineages, identified as β-tubulin-III positive for neurons and GFAP positive for astrocytes (Figure 1C and 1D).

### TMZ and MET combined to inhibit GSCs self-renewal and inhibit gliosphere formation and expansion

U87 GSCs and U251 GSCs sub-cultured in complete NBM, promoted gliosphere formation in 5–7 days and increased in size in 7–10 days. *In vitro* treatment with TMZ or MET resulted in a obvious decrease in the number and size of gliospheres generated from U87 and U251 glioma cells (*P* < 0.05). Interestingly, the combinatorial treatment with MTZ and MET showed more significant decrease in the number and size of gliospheres compare with either drug alone (*P* < 0.01) (Figure 2A and 2B). These results showed that the combined treatment of two drugs inhibits GSCs self-renewal capability. These results also suggested that combination treatment with TMZ and MET contribute more effectively to inhibit U87 and U251 secondary gliosphere formation and expansion compare with any single drug.

### MET synergistically enhances the inhibitory effects of TMZ on inhibiting glioma cell proliferation

**Figure 3:**
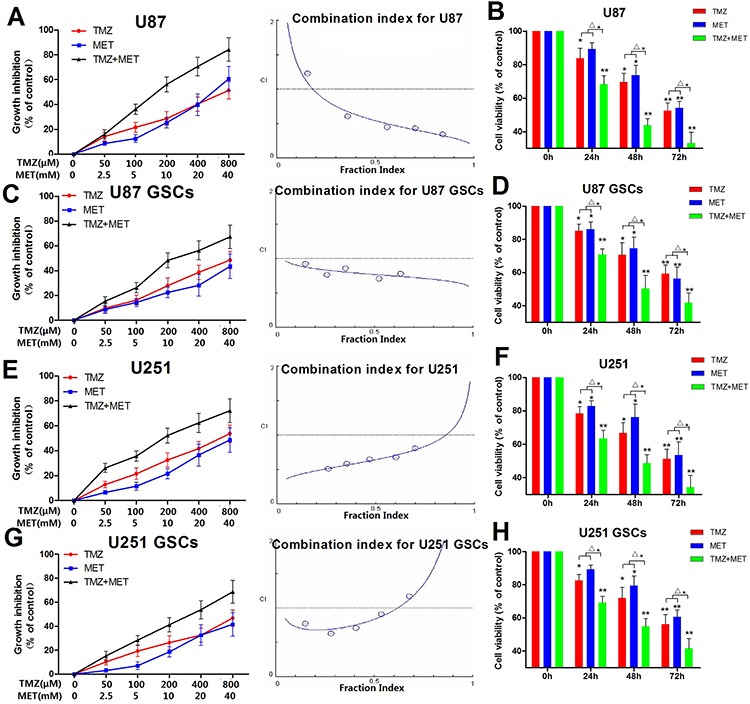
MET augments the effects of TMZ in glioma cell and GSC proliferation Cytotoxicity was detected by CKK-8 assay. **A, C, E, G.** U87, U87 GSCs, U251 and U251 GSCs were plated with different concentration of TMZ (0–800 μM), MET (0–40 mM) for 48 hours. **B, D, F, H.** U87, U87 GSCs, U251 and U251GSCs were treated with 200 μM of TMZ or/with 10 mM MET for different time points. The Fa-CI plot shows the combination index value (CI) for each fractional effect. The curves were generated using CalcuSyn software. A, C, E, G (Right panel) Results showed that TMZ had a synergistic effect with MET (CI < 1). (*P < 0.05, **P < 0.01, compared with control; ^Δ^*P < 0.05, TMZ+MET *vs* TMZ or MET).

In order to determine whether the MET currently in clinical trials for cancer treatment, can augment the cell proliferation inhibitory effects of TMZ in glioma cells and GSCs, glioma cells and GSCs (derived from U87 and U251 glioma cell lines) were plated with TMZ either alone or with MET, all cells were assessed using the CCK-8 assay. U87, U87GSCs, U251 and U251 GSCs showed a dose- and time-dependent response to proliferation inhibition by TMZ or/and MET (Figure 3). The combination of TMZ and MET (the doses of the TMZ and MET were at a 1:50 ratio of each other) resulted in a significant shift in the proliferation inhibition curve compared with either drug alone, caused a synergistic effect (*P* < 0.05, TMZ+MET *vs* single drug) (Figure 3B, 3D, 3F and 3H). The statistical combination index (CI) was determined for the dual therapy to determine whether combination therapy was synergistic, additive, or antagonistic. As shown in Figure 3A, 3C, 3E, 3G (right panel), MET acted synergistically (CI<1.0) with TMZ to inhibit U87, U87 GSCs, U251 and U251GSCs growth at almost all combination doses tested. These results showed that MET worked synergistically with TMZ to enhance the effects of either drug alone.

### Combined treatment of TMZ and MET treatment leads to GSCs apoptosis

**Figure 4:**
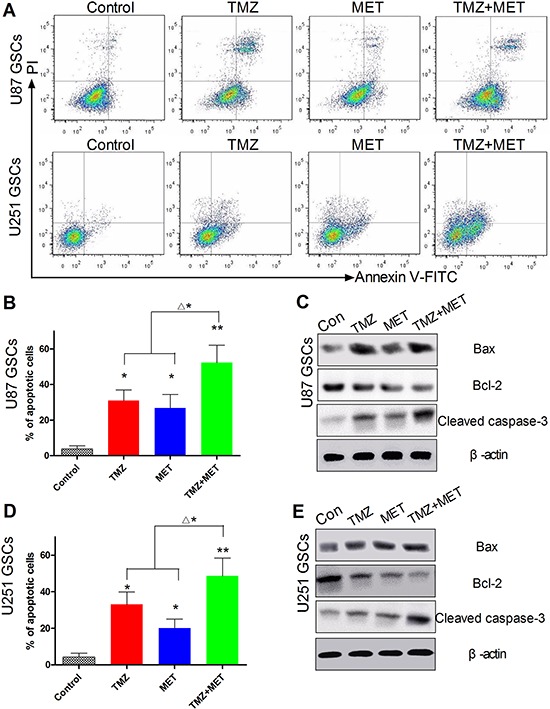
Effect of TMZ and MET on GSCs apoptosis **A.** Representative results of the synergistic effect of TMZ and MET for U87 GSCs and U251 GSCs. U87 GSCs and U251 GSCs were exposed to TMZ(200 μM), MET(10 mM), or the combination for 48 hours. Annexin V-positive cells were regarded as apoptotic cells. **B, D.** Quantitative analysis of the early and late apoptosis rate (*P < 0.05, **P < 0.01 compared with control group; ^Δ^*P < 0.05, TMZ+MET *vs* single drug). **C, E.** Western blots were performed to verify the combination effect for U87 GSCs and U251 GSCs. Compared with single drug, the GSCs treated with TMZ+MET, the protein levels of caspase-3 and Bax significantly increased, and Bcl-2 decreased.

To better understand the mechanism underlying the combined antiproliferative activity observed in the CCK-8 assays, GSCs were exposed to TMZ and MET, singly and in combination, and quantifying the percentage of apoptotic cells by annexin V/PI staining with flow cytometry. As shown in Figure 4A and 4B, combination treatment in U87 GSCs for 48 hours induced an increase in the percentage of apoptotic cells: 3.7 ± 1.9% for control, 31.0 ± 5.9% for TMZ, 26.8 ± 6.6% for MET and 52.3 ± 9.7% for TMZ and MET combined. Strikingly, the combinatorial treatment significantly inducing GSCs apoptosis compared with single drug (*P* < 0.05) (Figure 4B). In addition, the expression of Bcl-2 was significantly decreased, meanwhile the activities of Bax and cleaved caspase-3 were markedly elevated, compared with the TMZ or MET groups (Figure 4C). Similar results were observed in U251 GSCs (Figure 4A, 4D and 4E).

### The cell growth inhibitory effect of combinatorial treatment with TMZ and MET through AMPK but independent of AMPK

**Figure 5:**
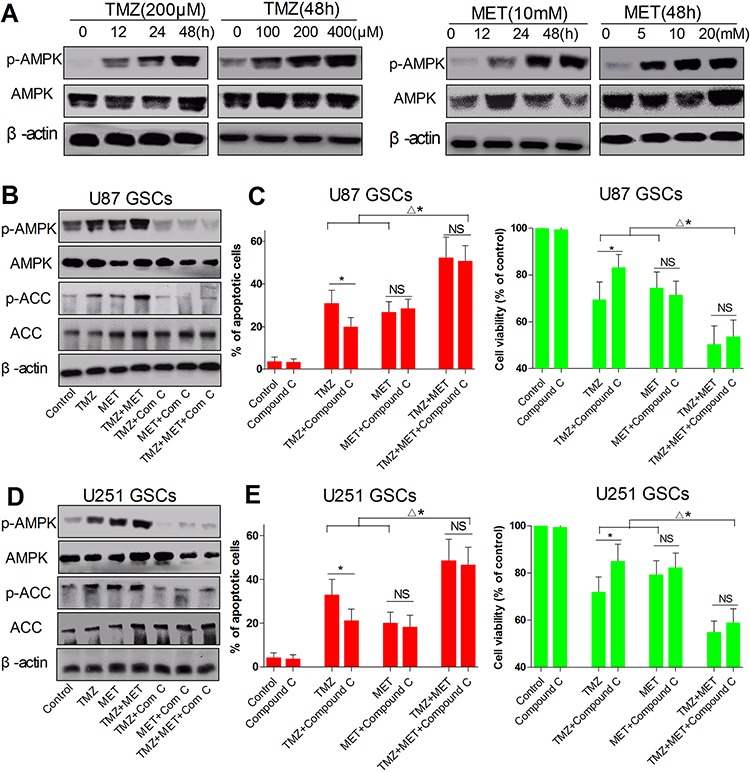
AMPK signaling in U87 GSCs and U251 GSCs upon TMZ and MET combinatorial treatment **A.** U87 GSCs was treated with TMZ (200 μM), MET(10 mM) and cultured for different time points (0, 12, 24, 48 hours) or treated with different doses of TMZ (0, 100, 200, and 400 μM), MET (0, 5, 10 and 20 mM) and cultured for 48 hours, AMPK and β-actin were detected by Western blotting. **B.** U87 GSCs and **D.** U251 GSCs were treated with TMZ (200 μM), MET (10 mM), compound C (2 μM) or their combination for 48 hours, AMPK, ACC and β-actin were detected by Western blotting. **C, E.** Cell viability was detected by CCK-8 assay (*P < 0.05, TMZ *vs* TMZ+compound C; ^Δ^*P < 0.05, TMZ + MET+compound C *vs* TMZ, MET, TMZ+compound C, MET+compound C; NS: no significant); The apoptosis cells were quantified (percentage) using an annexin V- FITC/PI apoptosis detection kit (*P < 0.05, TMZ *vs* TMZ+compound C; ^Δ^*P < 0.05, TMZ + MET+compound C *vs* TMZ, MET, TMZ+compound C, MET+compound C; NS: no significant).

MET, as an AMPK-activating agent, is widely used to suppress tumor cell proliferation. To examine whether the GSCs growth inhibitory effect of treatment with TMZ and MET is also mediated by activation of the AMPK signaling pathway, we conducted an immunoblots analysis using AMPK and ACC antibodies and parallel cell proliferation assays. As shown Figure 5A, in U87 GSCs, both TMZ and MET clearly induced AMPK phosphorylation in a dose-and time-dependent manner. As expected, immunoblots analysis showed obvious activation of phospho-status of AMPK and its downstream molecular ACC for GSCs under combination treatment as compared with single drug treatments (Figure 5B and 5D).

To further explore whether AMPK is involved in the synergistic effect from the combination treatment, AMPK activity was inhibited with its inhibitor compound C (2 μM). As shown in Figure 5C and 5E TMZ or/and MET treatment for 48 hours significantly suppressed cell proliferation and induced cell apoptosis in U87 GSCs and U251 GSCs. Interestingly, TMZ-induced cell apoptosis and death were largely repressed in AMPK inhibiting cells (*P* < 0.05, TMZ *vs* TMZ+compound C), but there was only a slight effect on MET (*P* > 0.05, MET *vs* MET+compound C) and the MET alone still induced a significant level of cell apoptosis and death (Figure 5B and 5C, 5D and 5E). Although AMPK activation inhibited by compound C, the combination treatment of TMZ and MET still produced synergistic cell apoptosis compared with any single drug. Moreover, compound C mildly inhibited TMZ- and MET-induced GSCs apoptosis (*P* > 0.05, TMZ+MET+compound C *vs* TMZ+MET) (Figure 5C and 5E). These findings along with the data from compound C (Figure 5B–5E) suggest that alteration of AMPK pathway may be one of the mechanisms for the synergism between TMZ and MET combination, but AMPK activation is not solely or definitively responsible for the synergistic effect for the combination of TMZ and MET.

### MET enhances the cytotoxicity of TMZ through downregulation of AKT-mTOR signaling pathway

**Figure 6:**
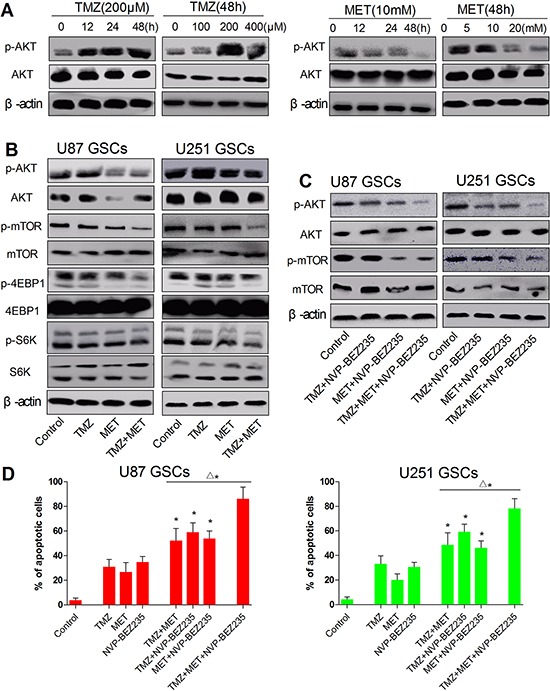
MET effectively reverse the AKT activation induced by TMZ, combined treatment with TMZ and MET synergistically reduces mTOR, S6K and 4EBP1 signaling **A.** U87 GSCs was treated with TMZ (200 μM), MET(10 mM) and cultured for different time points (0, 12, 24, 48 hours) or treated with different doses of TMZ (0, 100, 200, and 400 μM), MET (0, 5, 10 and 20 mM) and cultured for 48 hours, AKT and β-actin were detected by Western blotting. **B, C.** U87 GSCs and (E, G) U251 GSCs were treated with TMZ (200 μM), MET (10 mM), NVP-BEZ235 (20 nM) or their combination for 48 hours, p-AKT, p-mTOR, p-S6K, p-4EBP1 and β-actin were detected by Western blotting. **D.** the apoptosis cells were quantified (percentage) using an annexin V- FITC/PI apoptosis detection kit (*P < 0.05, compared with single drug; ^Δ^*P < 0.05, TMZ+MET+NVP-BEZ235 *vs* TMZ+MET, TMZ+NVP-BEZ235, MET+NVP-BEZ235).

It has proved that endogenous AKT can be activated in TMZ-treated cells [9], overexpression of an active form of AKT increases glioma cell resistance to TMZ [6] and inhibition of AKT function is associated with increased cell sensitivity to TMZ [9]. MET has been shown to exert antiproliferative activity on GSCs through inhibition of the AKT-mTOR pathway [24]. To explore whether MET augments the cytotoxicity of TMZ by downregulation of AKT-mTOR signaling pathway, we analyzed cell signaling changes after various drug treatments (TMZ, MET, TMZ and MET) in U87GSCs and U251 GSCs. As shown Figure 6A, in U87 GSCs, TMZ induced AKT phosphorylation in a time-dependent manner and MET decreased phosphorylation of AKT in a dose- and time-dependent manner. MET effectively reversed the AKT activation induced by TMZ after 48 hours treatment (Figure 6B). We although found that single-agent treatment with TMZ or MET showed obvious inhibition of phosphorylated mTOR, 4EBP1, and PS6K, under combination of the two drugs, the activated forms of these proteins were significantly suppressed under their combination (Figure 6B).

To further explore whether AKT-mTOR is involved in the synergistic effect from the combination treatment. We knocked down AKT-mTOR using NVP-BEZ235, a dual PI3K/mTOR inhibitor (20nM). We examined the ability of NVP-BEZ235 to potentiate cell apoptosis in response to TMZ, and MET either alone or in combination (Figure 6C). Compared with single drug, treatment with NVP-BEZ235 significantly increases cell apoptosis in combination with TMZ or MET under standard growth conditions after 48 hours (*P* < 0.05). In addition, we found that the combination of NVP-BEZ235 with both TMZ and MET effectively promoted extensive cell apoptosis within 48 hours in U87 GSCs and U251 GSCs (*P* < 0.05) (Figure 6C and 6D). All together, these results show that TMZ combined with MET synergize to inhibit GSCs proliferation through downregulation of AKT-mTOR signaling pathway, since addition of AKT-mTOR inhibitor such as NVPBEZ-235 promotes massive and rapid cell death.

### Metformin displays synergistic activity with TMZ *in vivo* xenograft models

**Figure 7:**
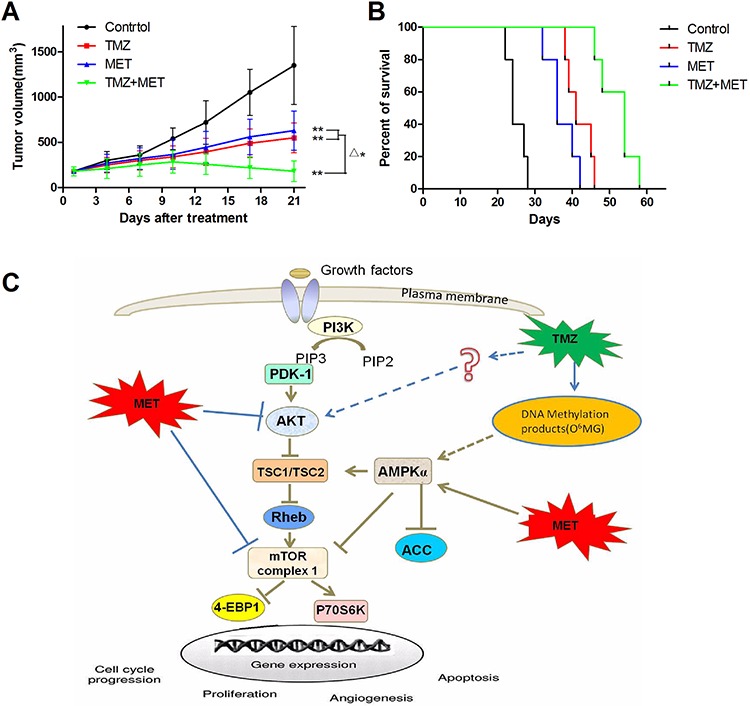
TMZ and MET synergize to inhibit glioma growth *in vivo* Subcutaneous tumors generated from U87 GSCs were allowed to reach a volume of 150–200 mm^3^ and were treated with TMZ (25mg/kg/day, intraperitoneal injection), MET (400 mg/kg/day, by gavage), or a combination of TMZ and MET. **A.** Tumor volumes in treatment groups. **B.** Survival of brain tumor-bearing mice was recorded and represented in a Kaplan–Meier plot. **C.** The proposed mechanism underlying the observed anti-glioma effect of TMZ and NVP-BEZ235. (**P < 0.01, compared with control group; ^Δ^*P < 0.05, TMZ + MET *vs* single drug).

We also examined whether the combination of MET and TMZ displays synergistically anti-glioma effects *in vivo*. Strikingly, combinatorial treatment with both drugs (TMZ+MET) resulted in reduced tumor growth rates (Figure 7A), the tumor volume for the control, TMZ alone (25 mg/kg, intraperitoneal injection), MET alone (400mg/kg, by gavage), and the combination of TMZ and MET at day 21 were 1350 ± 448 mm^3^, 550 ± 167 mm^3^, 630 ± 215 mm^3^, and 180 ± 114 mm^3^, respectively (*P* < 0.01). Encouragingly, combinatorial treatment significantly prolonged median survival of tumor bearing mice. As shown in the Kaplan-Meier curves (Figure 7B), the median survivals were 21, 41, and 36 days for the control, TMZ and MET groups, respectively (*P*< 0.01), whereas combined treatment extended median survival by 54 days(*P*< 0.05, TMZ+MET *vs* single drug). Figure 7C summarizes a possible network describing the principal mechanisms influenced by TMZ and MET.

## DISCUSSION

To date, no efficient and effective targeted therapies are available for GBM. The cancer stem cell theory has brought consciousness that eradicating cancer stem cells may overturn the drug-resistance after chemoradiation or targeted therapy [30, 31]. The catalog of documented genetic alterations found in GBM, with many occurring concurrently, suggests that single drug targeted therapies are unlikely to be effective in most patients [22, 32, 33]. In the present study, we focused on the synergistic mechanism of MET, an AKT inhibitor and AMPK agonist, to enhance the cytotoxicity of TMZ. We demonstrated for the first time that TMZ combined with MET more significantly inhibited GSCs growth both *in vitro* and *in vivo* compared with single drug treatment.

Our *in vitro* results demonstrated that the combination of TMZ and MET significantly inhibits glioma cells and GSCs proliferation, induce GSCs apoptosis and therefore, combination treatment significantly inhibits gliosphere formation and expansion. This inhibitory effect was time- and dose-dependent. Inhibition of anti-apoptotic Bcl-2 proteins can reduce apoptotic resistance in GBM [34]. Mechanistic studies using Western blot analysis revealed that NVP-BEZ235 could significantly reduce the expression of Bcl-2 in GSCs and activate Caspase-3 and Bax.

Cancer stem cells (CSCs) are a subpopulation of cells within a heterogeneous tumor. CSCs exhibit two essential properties: the capacity for self-renewal and the differentiation capacity [35]. It is generally agreed that the tumorsphere-forming cells possess cancer stem cell properties and are capable of self-renewal and display higher tumorigenicity. Self-renewal is assessed with *in vitro* tumorsphere formation assay, the sphere formation ability is evaluated over serial passaging, which is an indicator of long-term self-renewal [36]. In the present study, the formation of secondary gliospheres (a tumorsphere from GSCs) in control group indicated that GSCs had obvious self-renewal capability. The self-renewal capability of GSCs is assessed with secondary tumorsphere assays *in vitro* [37]. Importantly, we found that the combined treatment TMZ and MET significantly inhibited the secondary gliosphere formation of GSCs, suggesting that the combined treatment of two drugs inhibits synergistically GSCs serial self-renewal capability and reduces or partly eliminates GSCs.

Understanding the principal molecular mechanism underlying the inhibitory activity of TMZ/MET is pivotal to develop this combination as a novel therapy to decrease the risk of GBM relapse. AMPK is a metabolic-sensing protein kinase, which plays an critical role as an energy sensor mainly in regulating growth and reprogramming metabolism [38]. In the activated states, AMPK down-regulates several anabolic enzymes and thus inhibits cell growth [5]. Both TMZ and MET-induced GSCs death and apoptosis *in vitro* through AMPK activation, but the AMPK activation is not solely responsible for the synergistic effect for the combination of TMZ with MET. Although compound C-mediated suppression of AMPK inhibits TMZ-induced GSC apoptosis and death, but it's incomplete and compound C has little effect on MET-induced GSC death and apoptosis. In addition, after AMPK activation was inhibited by compound C, the combination of TMZ and MET still produced synergistic GSCs apoptosis compared with single agent, and the GSCs apoptosis rate without obvious falling compared with TMZ and MET group. Base on these results, we conclude that inhibition of AMPK activation suppresses TMZ-induced GSC apoptosis and death, thus we speculate that an increased in activity of AMPK enhances TMZ-induced GSC death, but not MET, a similar result has also been reported by Liu's [39] group.

AKT is a major downstream target of growth factor receptor tyrosine kinases that signal through PI3K. The activity of PI3K-AKT-mTOR pathway has consistently been linked to tumor cell resistance to antineoplastic agents [12, 40, 41]. In the present study, our results demonstrated that TMZ induced GSCs apoptosis via activating AMPK, but AKT kinase activity was activated at the same time. TMZ induced AKT activation, as well as deactivated mTOR. It has proved that inhibition of AKT expression was accompanied by a significant increased in glioma cell sensitivity to TMZ [9, 13]. These results strongly support the hypothesis that clinical benefits are obtained by combining TMZ with inhibitors of the AKT-mTOR pathway. AKT-mTOR pathway represents a new target for the sensitization of GBM to TMZ. MET effectively inhibited proliferation and induced apoptosis in GSCs, were associated with the inhibition of AKT-mTOR axis. For the combination treatments, MET enhanced the cytotoxicity of TMZ in GSCs, had a synergistic effect. Western blot results showed that MET effectively reversed the AKT activation induced by TMZ. Moreover, the activities of mTOR, 4EBP1 and S6P were significantly suppressed under their combination. To further explore whether AKT-mTOR is involved in the synergistic effect from the combination treatment. We knocked down AKT-mTOR using NVP-BEZ235, a dual PI3K-mTOR inhibitor. We demonstrate that inhibition of endogenous AKT-mTOR function sensitizes GSCs to TMZ and MET, addition of AKT-mTOR inhibitor such as NVPBEZ-235 promotes massive and rapid cell death. Here we speculate that the synergy between AKT-mTOR inhibition and MET may occur in other cancer.

Our *in vitro* study also demonstrated that TMZ in combination with MET treatment significantly reduces tumor growth rates and prolonged median survival of tumor-bearing mice.

To conclude, MET synergistically enhances the cytotoxicity of TMZ through down-regulation of the AKT-mTOR signal pathway, independent AMPK. This synergy was confirmed both *in vitro* and *in vivo*. This combination treatment could be a promising option for patients with advanced GBM.

## MATERIALS AND METHODS

### Reagents

Metformin was purchased from Sigma Chemical and dissolved in ultrapure water to obtain a stock concentration of 2000 mM; Temozolomide was purchased from Sigma Chemical and dissolved in dimethyl sulfoxide (DMSO, Sigma Aldrich) to obtain a stock concentration of 500 mM. Compound C, an AMPK inhibitor, was purchased from Sigma Chemical and dissolved in DMSO to obtain a stock concentration of 500 mM. NVP-BEZ235, a dual PI3K-mTOR inhibitor, was purchased from Selleck Chemicals and dissolved in DMSO to obtain a stock concentration of 10 mM. All reagents were aliquoted and stored at −80°C and diluted to the desired final concentration in DMEM at the time of use. The final concentration of DMSO was less than 0.1% in all the cell cultures and did not exert any detectable effect on cell growth or cell death.

### Cell culture

The human glioma cell lines U87 and U251 were purchased from American Type Culture Collection (ATCC). Both cell lines were cultured in DMEM media (Gibco, USA), supplemented with 10% fetal bovine serum (Hyclone, USA), Penicillin-Streptomycin(100 U/ml, Hyclone), glutamine(2 mM, Hyclone) in a humidified atmosphere of 5% CO_2_ at 37°C. The cells were dissociated using 0.25% trypsin and 0.02% EDTA solution and subcultured once in 2–3 days.

To generate GSCs, U87 and U251 glioma cells were dissociated from DMEM cultures using trypsin–EDTA solution and cultured in Neurobasal medium (NBM, Gibco) supplemented with N2 (1 ×, Gibco), B27 (1 ×, Gibco), glutaMAX (1 ×, Gibco), heparin (2 μ g/ml), recombinant human FGF-basic (b-FGF, 20 ng/ml, PeproTech), recombinant human epidermal growth factor (EGF, 20 ng/ml; PeproTech), Penicillin-Streptomycin (100 U/ml, Hyclone). The GSCs were cultured in 6-well plates in 5% CO_2_ incubator at 37°C with a medium change every 2–3 days. After gliospheres formed and reached 100–200 cells/sphere, within 10 days, gliospheres were dissociated by Accutase (Sigma) and reseeded at a ratio of 1:2–3.

### GSCs identification (Immunofluorescence staining)

The secondary gliospheres were plated onto poly-L-lysine (Sigma) coated glass cover slips in DMEM with 10% FBS for 8 hours. The Gliospheres were washed with cold PBS, fixed with 4% paraformaldehyde for 30 min, permeabilized with 0.1% Triton X-100 for 15 min and blocked in 5% BSA (Sigma) for 1 hour at room temperature. Then the gliospheres were immunostained with CD133 (1:100, ZSGB-BIO, China), nestin (1:40, ZSGB-BIO, China), β-tubulin III (1:100, ZSGB-BIO, China) and glial fibrillary acidic protein (GFAP, 1:100, ZSGB-BIO, China) at 4°C overnight. Subsequent visualization was performed with fluorochrome-conjugated secondary antibody (ZSGB-BIO, China) for 0.5 hours at room temperature in darkness, and the nuclei were counterstained with DAPI.

For immunostaining of differentiated tumor cells, gliospheres were tanslated to DMEM with 10% FBS for another 7 days, immunocytochemistry was performed as described above. The fluorescent signals were detected and photographed with a fluorescence microscope (Olympus IX51, Japan).

### Gliosphere formation and expansion assay

To test the effect of TMZ or/and MET on secondary gliosphere formation, after primary spheres formation was noted, the primary gliospheres were dissociated and plated in 96-well plates (5 × 10^3^ per ml per well) in NBM in the absence or presence of TMZ, MET or TMZ + MET, Cultures were fed 0.02 ml of NBM every 2 days and photographed (× 200) after 7 days using IX51 Olympus microscope.

### Cell proliferation assays and synergy analysis

The cytotoxicity of TMZ or MET on glioma cells was determined by cell counting kit-8 (CCK-8) assay. Cells in the logarithmic growth phase were seeded in 96-well microplates at a density of 5 × 10^3^ cells in 200 μl media per well and incubated for 24 hours prior to treatment, then different concentrations of TMZ or MET were added and compared with the DMSO-treated control. Twenty microliters of CCK-8 solution were added 4 hours before the end of the incubation period, and the OD was measured at 450nm after 72 hours using a Synergy HTX Multi-Mode Microplate Reader (BioTek, USA).

To determine the dose-effect of combination therapy (TMZ plus MET) at 48 hours of treatment, the Chou-Talalay method and CalcuSyn software (version 2, Biosoft, Cambridge, UK) were used [42]. For this synergy analysis, TMZ was combined with MET at a constant ratio for glioma cells and GSCs at a dosage determined by the IC_50_ of each drug. Interaction was quantified based on a combination index (CI) to assess synergism (CI < 1), additive effect (CI = 1), and antagonism (CI > 1).

### Apoptosis assays

The apoptosis cells were quantified (percentage) using an Annexin V-fluorescein isothiocyanate (FITC)/propidium iodide (PI) apoptosis detection kit (BD). Briefly, cells in the logarithmic growth phase were seeded at a density of 2 × 10^5^ cells per well in 6-well plates. After treatment, cells were harvested using Accutase detachment solution and Annexin-V-FITC/PI labeling was performed according to the manufacturers’ instructions. The stained cells were analyzed with a flow cytometer. The numbers of viable (annexin V−/PI-), apoptotic (annexin V+/PI-), and necrotic (annexin V+/PI+) cells were calculated with the FACSDiva Version 6.2.

### Western blotting

GSCs were seeded in 100 mm dishes (Falcon) and incubated for 24 hours prior to treatment. After treatment, GSCs were collected and lysed in RIPA buffer, and centrifuged at 12 000 × g for 15 min. Supernatants were collected, and the total protein concentration was quantified using the bicinchoninic acid (BCA) assay kit. Equal amounts of proteins (30 μg) were separated by SDS-PAGE gels and transferred to a PVDF membrane. After blocking with 5% skim milk at room temperature for 2 h, the membranes were incubated with primary antibodies against rabbit anti-active caspase-3, rabbit anti-BAX, rabbit anti-Bcl-2, rabbit antiphospho-AMPK, rabbit anti-AMPK, rabbit antiphospho-acetyl CoA carboxylase (ACC), rabbit anti-ACC, rabbit antiphospho-AKT, rabbit anti-AKT, rabbit antiphospho-mTOR, rabbit anti-mTOR, rabbit antiphospho-4EBP1, rabbit anti-4EBP1, rabbit antiphospho-p70S6K, rabbit anti-p70S6K (All of the above antibodies were procured from Cell Signaling Technology), equal lane loading was confirmed using a monoclonal antibody against β-actin (Sigma-Aldrich). The membranes were washed three times with PBS-T (0.1% (v/v) Triton-X100) buffer for 0.5 hours and incubated with HRP-conjugated secondary antibody for 2 hours. After washing with the PBS-T buffer, the membranes were scanned with the Odyssey Infrared Imaging System (LI-COR).

### Human tumor xenografts in severe combined immunodeficient (SCID) mice

To test the anti-glioma effect of TMZ alone or in combination with MET *in vivo*, a xenograft model of human glioma was established. 4-week old male SCID mice were purchased from Vital River Laboratory Animal Technology Co. Ltd. (Beijing, China). After 1 week acclimatization, each mouse was injected subcutaneously in the right flank with 1 × 10^6^ U87 GSCs resuspended in 50 μl NBM media. After about 3 weeks, when the subcutaneous tumors reached an average size of 150 to 200 mm^3^, mice were randomly divided into 4 groups (five mice per group) and treatment was started. Mice received TMZ alone (25 mg/kg/day, for a 21-day continuum, intraperitoneal injection) or MET alone (400 mg/kg/day, for a 21-day continuum, by gavage) or TMZ and MET in combination or vehicle as control. Tumor diameter was measured every 2–3 days with calipers, and the tumor volume was calculated (length × width × width × 0.5). The survival time of the mice was recorded and the median survivals were calculated. All animal procedures were approved by the Animal Ethics Committee, First Hospital of Jilin University, Changchun, China. All surgery was performed under sodium pentobarbital anesthesia, and all efforts were made to minimize suffering.

### Statistical analysis

All experiments were performed intriplicate unless otherwise noted, and results were expressed as mean ± standard deviation. The *P*-values less than 0.05 were considered statistically significant. All of the analyses were performed with GraphPad Prism 5.0.
